# Detection of Genomic Imprinting for Carcass Traits in Cattle Using Imputed High-Density Genotype Data

**DOI:** 10.3389/fgene.2022.951087

**Published:** 2022-07-15

**Authors:** David Kenny, Roy D. Sleator, Craig P. Murphy, Ross D. Evans, Donagh P. Berry

**Affiliations:** ^1^ Animal and Grassland Research and Innovation Centre, Teagasc, Moorepark, Co. Cork, Ireland; ^2^ Department of Biological Sciences, Munster Technological University, Bishopstown Campus, Co. Cork, Ireland; ^3^ Irish Cattle Breeding Federation, Highfield House, Bandon, Co. Cork, Ireland

**Keywords:** genomic imprinting, carcass traits, epigenetics, association analysis, non-additive

## Abstract

Genomic imprinting is an epigenetic phenomenon defined as the silencing of an allele, at least partially, at a given locus based on the sex of the transmitting parent. The objective of the present study was to detect the presence of SNP-phenotype imprinting associations for carcass weight (**CW**), carcass conformation (**CC**) and carcass fat (**CF**) in cattle. The data used comprised carcass data, along with imputed, high-density genotype data on 618,837 single nucleotide polymorphisms (**SNPs**) from 23,687 cattle; all animal genotypes were phased with respect to parent of origin. Based on the phased genotypes and a series of single-locus linear models, 24, 339, and 316 SNPs demonstrated imprinting associations with CW, CC, and CF, respectively. Regardless of the trait in question, no known imprinted gene was located within 0.5 Mb of the SNPs demonstrating imprinting associations in the present study. Since all imprinting associations detected herein were at novel loci, further investigation of these regions may be warranted. Nonetheless, knowledge of these associations might be useful for improving the accuracy of genomic evaluations for these traits, as well as mate allocations systems to exploit the effects of genomic imprinting.

## Background

Genetic selection has translated into performance gains in farmed livestock globally (Havenstein et al., 2003; Twomey et al., 2020). The calculation of genetic merit estimates, and the downstream ranking of sires based on their estimated genetic merit, through genetic evaluations, has historically used a combination of phenotypic and pedigree information (Wiggans et al., 1988; Graser et al., 2005; Kenny et al., 2020a). The incorporation of genomic information into genetic evaluations in the past two decades has accelerated the rate of genetic gain (García-Ruiz et al., 2016; Xu et al., 2020). This approach to genetic evaluations assumes that performance and inter-animal genetic differences are due to differences in the DNA sequences of the animal’s genome. The epigenome deviates from this assumption through a potential change in an animal’s gene expression and phenotypic performance without an alteration in the underlying DNA sequence (González-Recio et al., 2015).

Of interest to the present study is the epigenetic phenomenon known as genomic imprinting. Genomic imprinting is where the expression of an allele differs depending on the sex of the transmitting parent (Reik and Walter, 2001). While the underlining mechanisms of genomic imprinting are both numerous and complex (MacDonald, 2012), they can manifest themselves in the form of either complete imprinting (i.e., where the allele inherited from one parent is not expressed) or partial imprinting (i.e., where the paternally inherited allele is expressed differently to the same maternally inherited allele). Since the phenomenon of parent-specific allele expression was first documented in mice by Cattanach and Kirk (1985), further examples of the phenomenon have been documented in a variety of species including pigs, sheep and cattle (Essl and Voith, 2002; Georges et al., 2003; Guo et al., 2016). Knowledge of the presence of genomic imprinting has previously been shown to be beneficial in selection programs, as, complementary to selection based on additive genetic merit, parents could be selected to exploit (or avoid) the effects of genomic imprinting on the performance of their resulting progeny (González-Recio et al., 2015). Therefore, genomic imprinting can potentially be exploited in mating advice programs. Additionally, consideration of imprinting effects in genomic evaluations has been reported to improve the accuracy of additive genomic breeding values (Nishio and Satoh, 2015).

For carcass traits in cattle (i.e., the traits of interest to the present study), genomic imprinting has been reported to account for 8–39% of the total additive genetic variance (Neugebauer et al., 2010; Inoue et al., 2021); while imprinting is a non-additive effect, when not accounted for, imprinting can be confounded with additive effects. The objective of the present study was to detect the presence of genomic imprinting for beef carcass traits [i.e., carcass weight (**CW**), carcass conformation (**CC**) and carcass fat (**CF**)] using phased (with respect to parental origin), high-density genotype data across the genome. Although Imumorin et al. (2011) previously reported the presence of quantitative trait loci with parent-of-origin effects on cattle carcass traits, namely CW and kidney, pelvic and heart fat, their analyses was based on a linkage map constructed using 357 microsatellites, as opposed to the high-density single nucleotide polymorphism (**SNP**) data used herein. Results from the present study should provide further insight into the epigenetic components underlying the carcass merit of cattle. Furthermore, such information could potentially be used to improve the accuracy of genomic evaluations and to inform mate selection programs.

## Methods

### Genomic Data

Previously imputed high-density genotypes, comprising 734,159 autosomal SNP genotypes from 638,662 cattle were available. An in-depth description of the pipeline used to impute the genotype data to high density is provided by Purfield et al. (2019b). All 638,662 animals had been genotyped on one of the following panels: the Illumina HD panel (777,962 SNPs), the Illumina Bovine SNP50 panel (54,001 SNPs), or one of the custom Irish Dairy and Beef (IDB) genotype panels, namely the IDBV1 (16,662 SNPs), the IDBV2 (16,223 SNPs) or the IDBV3 (52,445 SNPs) panels. Imputation to high density was conducted for all genotyped animals using a two-step approach in FImpute2 (Sargolzaei et al., 2014). Only autosomal SNPs with a known chromosome and position were considered for imputation. In addition, all imputed animals and SNPs had a call rate ≥90%. The first step in the imputation process involved imputing animals genotyped on the IDB genotype panels to the Illumina Bovine SNP50 density. All animals with genotype information at the Bovine SNP50 density (imputed or not), were then imputed to high density using a multi-breed reference population of 5,504 high-density genotyped males specifically targeted for genotyping given their contribution to the Irish cattle population.

### Phenotypic Data

The three carcass phenotypes of interest to the present study were CW, CC, and CF, all of which were measured in accordance with the EUROP grading system. Carcass weight is measured in kg, on average, one hour after slaughter, following the removal of the head, hide, legs, thoracic and abdominal organs, and internal fat. With regard to CC and CF, the former reflects the shape and development of the carcass, particularly on the round, back and shoulders, and the latter reflects the level of fat covering the carcass, as well as within the thoracic cavity of the carcass (Kenny et al., 2020b). Under the EUROP grading system, CC scores are represented by the letter E (best), U, R, O, and P (worst), which are subdivided into three subscores (i.e., −, = and +). Carcass fat scores are represented by the numbers 1 (lowest fat cover), 2, 3, 4, and 5 (highest fat cover), with the same three subscores applied (i.e., −, = and +).

Only genotyped young bulls, heifers and steers slaughtered between the ages of 12 and 36 months, inclusive, with recorded carcass phenotypes, of which there was 93,470 animals, were considered for the present study. Additionally, all young bulls, heifers and steers considered were born to dams with a parity number ≤10 and were not born from embryo transfer. Finally, any cattle with more than three inter-herd movements during their life, or a movement 100 days before slaughter were not considered further. Following all edits, the birth herd of all remaining genotyped animals were categorized as either beef or dairy based on parameters outlined by Ring et al. (2018). Herds were classified as beef when the average dam breed composition within the herd consisted of ≤65% dairy breeds (i.e., Holstein-Friesian or Jersey), whereas, herds were classified as dairy when the average dam breed composition consisted of >75% dairy breeds. Any animals born in herds that remained unclassified were not considered further. After edits, 73,040 genotyped animals remained. All remaining genotyped animals were allocated to herd-year-sex contemporary groups, comprising animals of the same sex that were slaughtered from the same herd within 60 days of one another, using an algorithm which is routinely used in the Irish genetic evaluations (Pabiou et al., 2012). Only animals in contemporary groups containing at least five animals were retained. After all edits, 23,687 genotyped animals with carcass phenotypes remained. For all animals, the sire and dam was known and, for the purpose of genotype phasing, at least one of each animal’s parents had (imputed) high-density genotype data available. Using the genotype data of the 23,687 animals of interest, SNPs with minor allele frequencies ≤0.05 were discarded. After quality control edits, 618,837 SNPs remained, each of which were based on ARS-UCD 1.2 genome build.

### Genotype Phasing

For each of the 23,687 animals of interest, the parental origin of each allele was inferred by phasing their genotypes using a combination of pedigree and genotype information in FImpute2 (Sargolzaei et al., 2014). The pedigree file constructed for the purpose of phasing included 130,178 genotyped animals and had a depth of five generations. The (imputed) high-density genotype data of all 130,178 animals were included in the phasing process.

Following phasing, four unique genotypes were distinguishable at each locus (e.g., AA, AB, BA and BB; where, in the case of the heterozygotes, the first allele is paternally inherited and the second allele is maternally inherited). For the purpose of the association analyses, the four genotypes were coded to represent additive [i.e., 0 (AA), 1 (AB and BA) and 2 (BB)], dominance [i.e., 0 (AA and BB) and 1 (AB and BA)] and imprinting effects [0 (AB), 1 (AA and BB) and 2 (BA)] (Supplementary Table S1).

### Genome-Wide Association Analysis

Based on the GRAMMAR method proposed by Aulchenko et al. (2007a), all carcass phenotypes were pre-adjusted for nuisance variables, fitted as fixed effects, as well as for the direct polygenic effect of the animals *via* a genomic relationship matrix. All phenotypes were adjusted in the GenABEL package in R (Aulchenko et al., 2007b) using the following model:

y=1μ+Xβ+Zα+e

where y was a vector of CWs, CC scores or CF scores; 1 was a vector of ones; μ was the population mean; β was a vector of fixed effects that included contemporary group, birth herd type (i.e., beef or dairy), dam parity and whether the animal was born a singleton or twin; α was a vector of random polygenic effects; e was a vector of random residual effects, and X and Z were incidence matrices for the fixed and random effects, respectively. The distribution of the random polygenic effect was assumed as $\;\alpha ∼N\left(0,G\sigma_{a}^{2}\right)$ , where G and ${\text{σ}}_{\text{a}}^{2}$ are, respectively, the genomic relationship matrix and the genetic variance. The genomic relationship matrix was constructed based on Method I described by VanRaden (2008). The distribution of the random residual effect was assumed as $e∼\text{ N}\left(0,I{\text{σ}}_{\text{e}}^{2}\right)$ ), where I and $\sigma_{\text{e}}^{2}$ are an identity matrix and the residual variance, respectively.

Association analyses were then conducted for each locus separately using the pre-adjusted carcass phenotypes as:

$$e=1\text{μ}+{\text{b}}_{1}a_{k}+{\text{b}}_{2}d_{k}+{\text{b}}_{3}i_{k}+e_{k}$$

where e is the vector of residuals for CW, CC or CF from the preceding model; 1 was a vector of ones; μ was the population mean; $a_{k}$ was the vector of additive genotype codes for locus k ; $d_{k}$ was the vector of dominance genotype codes for locus k ; $i_{k}$ was the vector of imprinting genotype codes for locus  k ; ${\text{b}}_{1}$ ${\text{b}}_{2}\;$ and ${\text{b}}_{3}\;$ were the regression coefficient associated with the additive, dominance and imprinting genotype codes, respectively, and $e_{k}$ were the residuals. Of particular interest in the present study was the significance of the ${\text{b}}_{3}$ model solutions from zero; it should be noted, that the solutions for ${\text{b}}_{3}$ represent the difference between the mean of the two (phased) heterozygous genotypes of a given locus and the mid-point of the two means of the corresponding homozygous genotypes (Wolf et al., 2008a). To test the significance of the ${\text{b}}_{3}$ regression coefficients and to correct for multiple testing, all *p* values for ${\text{b}}_{3}$ were transformed into q values (Storey and Tibshirani, 2003). All SNPs with a q value ≤0.01 were considered significant, while all SNPs with a q value >0.01 and ≤0.05 were considered suggestive.

### Quantitative Trait Loci Regions

Quantitative trait loci (**QTL**) regions associated with CW, CC, and CF were defined based on the flanking linkage disequilibrium patterns around the SNPs with significant or suggestive associations. To estimate the start and end positions of the QTL regions, all SNPs within a 0.5 Mb window that were in linkage disequilibrium (*r* ^2^ of ≥0.5) with significantly or suggestively associated SNPs were considered part of a single QTL region. In the case where QTL regions overlapped, these regions were merged together and considered a single region. Additionally, the presence of candidate genes within a 0.5 Mb window of the QTL regions, as well as genes located within the QTL regions were investigated using ENSEMBL (https://www.ensembl.org/) on the ARS-UCD 1.2 genome build, alongside the biomaRt package in R (Durinck et al., 2009). Furthermore, the geneimprint database (http://www.geneimprint.com; date last accessed 5 August 2021) were inspected to identify known imprinted genes within a 0.5 Mb window, either upstream or downstream, of the SNPs of interest.

## Results

### Carcass Weight

**FIGURE 1:**
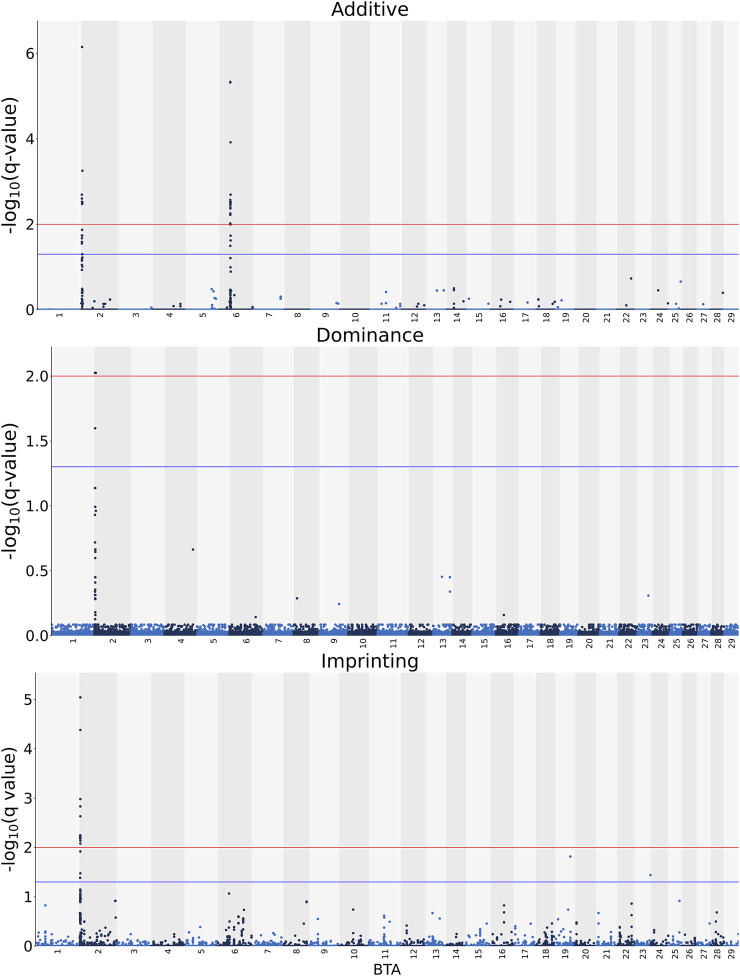
Manhattan plots of the −log_10_ (q values) for additive, dominance and imprinting associations with carcass weight from each *Bos taurus* (BTA) chromosome. Blue and red lines are the thresholds for suggestive (0.01 < q value ≤0.05) and significant (q values ≤0.01) SNPs, respectively.

The associations between CW and the additive, dominance and imprinting genotype codes of each SNP are shown in Figure 1. Furthermore, details of the imprinted QTL regions defined for CW are outlined in Table 1, with the names of genes co-located with each QTL provided in Table 2. Based on a genome-wide significance threshold of q ≤ 0.01, 19 SNPs, all located on *Bos taurus* (**BTA**) chromosome 2, demonstrated imprinting associations with CW. In addition, a further five SNPs located on BTA 2 (three SNPs), BTA 19 (one SNP) and BTA 23 (one SNP) demonstrated suggestive imprinting associations (0.01 < q ≤ 0.05) with CW. Based on the 24 SNPs demonstrating an imprinting association with CW, two distinct QTL regions were identified. These QTL regions stretched from 2.60 to 2.65 Mb on BTA 2 and from 3.43 to 3.71 Mb on BTA 2. Both lead SNPs (i.e., the SNP with the strongest association) in the QTL regions were intergenic variants, with no currently known imprinted gene residing within a 0.5 Mb window of the QTL regions.

**TABLE 1:** Chromosome (BTA), start and end position (bp), and number of significant and suggestive single nucleotide polymorphisms (SNPs) within the imprinted QTLs associated with carcass weight, as well as SNP name, position, imprinting effect, *p*-value, nearest gene within 0.5 Mb and annotation of the lead SNP.

BTA	QTL region			Lead SNP in QTL region					
	Start	End	SNPs	SNP name	Position	Effect	*p*-value	Gene	Annotation
2	2,602,551	2,644,497	2	rs134356704	2,644,497	0.986	7.03 × 10^−8^	-	Intergenic
2	3,434,078	3,712,235	17	rs110421260	3,699,886	1.314	2.34 × 10^−11^	HS6ST1	Upstream

**TABLE 2:** Chromosome (BTA) and start and end position (bp) of the imprinted QTL regions defined for carcass weight, as well as the name of genes located within a 0.5 Mb window of each region [distance (in Kb) from the lead SNP of each region in parenthesis]^a^.

BTA	Start	End	Genes
2	3,434,078	3,712,235	HS6ST1 (475)

a QTLs with no gene located within a 0.5 Mb window of their boundaries are not included in the table.

### Carcass Conformation

**FIGURE 2:**
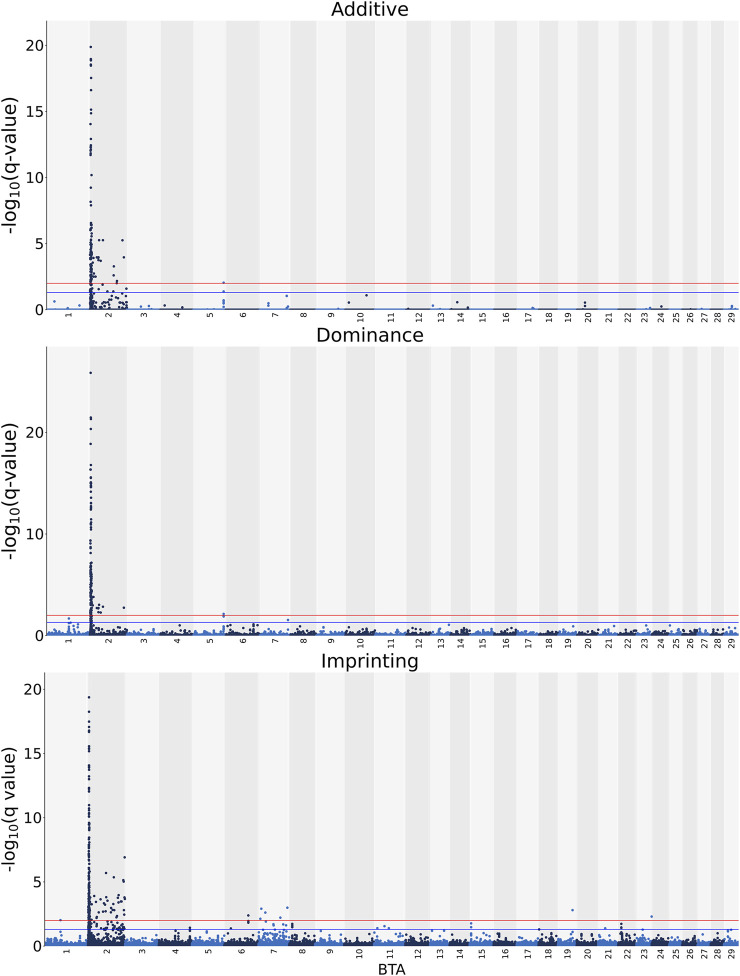
Manhattan plots of the −log_10_ (q values) for additive, dominance and imprinting associations with carcass conformation from each *Bos taurus* (BTA) chromosome. Blue and red lines are the thresholds for suggestive (0.01 < q value ≤0.05) and significant (q values ≤0.01) SNPs, respectively.

Manhattan plots with the additive, dominance, and imprinting associations between each of the 618,837 SNPs of interest and CC are presented in Figure 2. Details of the imprinted QTL regions defined for CC are provided in Table 3, with the names of co-located genes provided in Table 4. A total of 221 and 118 SNPs had significant and suggestive imprinting associations with CC, respectively. On BTA 2, 212 SNPs demonstrated significant imprinting associations with CC, while a further 95 SNPs, also located on BTA 2, demonstrated suggestive imprinting associations. Based on the SNPs demonstrating imprinting associations with CC located on BTA 2, 22 imprinted QTL regions, ranging from 0.9 to 390.6 Kb in length, were identified. Of the remaining nine SNPs demonstrating significant imprinting associations with CC, one was on BTA 1, one was on BTA 6, five were on BTA 7, one was on BTA 19 and one was located BTA 23. Furthermore, the 23 SNPs demonstrating suggestive imprinting associations with CC that were not located on BTA 2 were located on eight different chromosomes, namely BTA 4, 6, 7, 8, 11, 15, 21, and 22. Apart from the QTL regions identified on BTA 2, single imprinted QTL regions for CC were identified on BTA 6, 8, and 22 (Table 3). Of the different QTL regions identified, the associative lead SNPs included intronic variants of the genes: NIPA1, NIAP2, ARHGEF4, UGGT1, AMMECR1L, MYO7B, BIN1, DDX58, and STAC, as well as intergenic variants (Table 3). Nonetheless, no known imprinted gene resided within a 0.5 Mb window of the SNPs demonstrating imprinting associations with CC.

**TABLE 3:** Chromosome (BTA), start and end position (bp), and number of significant and suggestive single nucleotide polymorphisms (SNPs) within the imprinted QTLs associated with carcass conformation, as well as SNP name, position, imprinting effect, *p*-value, nearest gene within 0.5 Mb and annotation of the lead SNP.

BTA	QTL region			Most strongly associated SNP in each QTL					
	Start	End	SNPs	SNP name	Position	Effect	*p*-value	Gene	Annotation
2	970,896	999,035	4	rs137294745	999,035	−0.028	4.05 × 10^–7^	NIPA1	Intron
2	1,001,853	1,005,580	3	rs43598048	1,005,580	0.029	2.49 × 10^–7^	NIPA2	Intron
2	1,807,237	1,812,806	2	rs134649609	1,807,237	−0.039	1.46 × 10^–8^	ARHGEF4	Intron
2	1,928,840	2,073,647	7	rs134802628	2,073,647	0.045	7.70 × 10^–8^	PLEKHB2	Downstream
2	2,152,312	2,214,590	2	rs134827178	2,152,312	0.025	1.10 × 10^–5^	PLEKHB2	Downstream
2	2,324,118	2,555,551	14	rs110703910	2,423,609	0.042	3.97 × 10^–12^	PLEKHB2	Downstream
2	2,589,767	2,826,308	27	rs133122826	2,589,767	0.044	1.08 × 10^–12^	-	Intergenic
2	3,020,151	3,061,170	9	rs109992924	3,031,217	0.052	4.73 × 10^–15^	-	Intergenic
2	3,227,977	3,311,963	13	rs109083210	3,311,963	0.047	3.02 × 10^–15^	-	Intergenic
2	3,325,739	3,716,374	40	rs110005217	3,494,838	0.060	7.13 × 10^–26^	-	Intergenic
2	3,789,255	3,844,348	7	rs135042814	3,822,439	0.029	6.45 × 10^–6^	HS6ST1	Upstream
2	3,864,717	3,865,578	2	rs137199723	3,864,717	0.028	1.05 × 10^–5^	HS6ST1	Upstream
2	3,896,532	3,909,497	2	rs109255152	3,909,497	−0.033	1.25 × 10^–8^	HS6ST1	Upstream
2	3,952,949	3,954,934	2	rs135927481	3,954,934	0.025	1.08 × 10^–5^	HS6ST1	Upstream
2	4,056,116	4,144,803	9	rs132929511	4,095,906	−0.046	2.42 × 10^–14^	HS6ST1	Upstream
2	4,232,890	4,405,833	25	rs43290702	4,335,970	0.045	1.83 × 10^–14^	UGGT1	Intron
2	4,424,778	4,427,108	3	rs137671107	4,424,778	0.034	4.36 × 10^–9^	UGGT1	Downstream
2	4,442,970	4,525,365	16	rs110389976	4,454,604	0.031	1.15 × 10^−7^	SAP130	Upstream
2	4,611,946	4,615,194	2	rs109435427	4,615,194	0.028	4.45 × 10^−6^	AMMECR1L	Intron
2	4,970,559	4,975,993	4	rs136954804	4,970,559	0.023	2.07 × 10^−5^	MYO7B	Intron
2	5,429,764	5,442,744	2	rs136132036	5,442,744	0.072	6.54 × 10^−6^	BIN1	Intron
2	10,426,413	10,432,722	2	rs134368002	10,426,413	0.037	1.51 × 10^−5^	FSIP2	Upstream
6	84,171,205	8,4195,787	3	rs133471939	84,171,205	0.054	1.42 × 10^−6^	YTHDC1	Downstream
8	11,601,528	11,605,893	3	rs136047404	11,601,528	0.029	1.09 × 10^−5^	DDX58	Intron
22	10,225,287	10,229,539	2	rs109346239	10,208,890	−0.023	1.78 × 10^−5^	STAC	Intron

**TABLE 4:** Chromosome (BTA) and start and end position (bp) of each imprinted QTL regions defined for carcass conformation, as well as the name of genes located within a 0.5 Mb window of each region [distance (in Kb) from the lead SNP of each region in parenthesis]^a^.

BTA	Start	End	Genes
2	970,896	999,035	OCA2 (−266), HERC2 (−19), NIPA1 (0), NIPA2 (46), CYFIP1 (68), TUBGCP5 (234), CCDC115 (315), IMP4 (319), PTPN18 (324), AMER3 (475)
2	1,001,853	1,005,580	OCA2 (−297), HERC2 (50), NIPA1 (0), NIPA2 (39), CYFIP1 (61), TUBGCP5 (227), CCDC115 (309), IMP4 (313), PTPN18 (317), AMER3 (468)
2	1,807,237	1,812,806	CCDC115 (−489), IMP4 (−484), PTPN18 (−455), AMER3 (−331), ARHGEF4 (0), FAM168B (53), PLEKHB2 (107)
2	1,928,840	2,073,647	AMER3 (−452), ARHGEF4 (−64), FAM168B (−22), PLEKHB2 (0)
2	2,152,312	2,214,590	ARHGEF4 (−288), FAM168B (−246), PLEKHB2 (−182)
2	2,324,118	2,555,551	ARHGEF4 (−459), FAM168B (−417), PLEKHB2 (−354)
2	3,325,739	3,716,374	HS6ST1 (471)
2	3,789,255	3,844,348	HS6ST1 (343), UGGT1 (456)
2	3,864,717	3,865,578	HS6ST1 (322), UGGT1 (435)
2	3,896,532	3,909,497	HS6ST1 (278), UGGT1 (391)
2	3,952,949	3,954,934	HS6ST1 (232), UGGT1 (346), SAP130 (499)
2	4,056,116	4,144,803	HS6ST1 (43), UGGT1 (156), SAP130 (310), AMMECR1L (456), POLR2D (478)
2	4,232,890	4,405,833	HS6ST1 (−5), UGGT1 (0), SAP130 (49), AMMECR1L (195), POLR2D (218), WDR33 (271), SFT2D3 (385), LIMS2 (436), GPR17 (467), MY07B (481)
2	4,424,778	4,427,108	HS6ST1 (−198), UGGT1 (26), SAP130 (28), AMMECR1L (174), POLR2D (196), WDR33 (250), SFT2D3 (363), LIMS2 (415), GPR17 (446), MY07B (460)
2	4,442,970	4,525,365	HS6ST1 (−216), UGGT1 (−44), SAP130 (0), AMMECR1L (75), POLR2D (98), WDR33 (152), SFT2D3 (265), LIMS2 (317), GPR17 (347), MY07B (362)
2	4,611,946	4,615,194	HS6ST1 (−384), UGGT1 (−212), SAP130 (−82), AMMECR1L (0), POLR2D (8), WDR33 (62), SFT2D3 (175), LIMS2 (227), GPR17 (258), MY07B (271), IWS1 (416), PROC (487)
2	4,970,559	4,975,993	SAP130 (−440), AMMECR1L (−350), POLR2D (−333), WDR33 (−185), SFT2D3 (−179), LIMS2 (−83), GPR17 (−92), MY07B (0), IWS1 (55), PROC (126), MAP3K2 (223), ERCC3 (273), CYP27C1 (321), BIN1 (442)
2	5,429,764	5,442,744	MY07B (−440), IWS1 (−372), PROC (−317), MAP3K2 (−195), ERCC3 (−150), CYP27C1 (−110), BIN1 (0), NAB1 (192), NEMP2 (343), MFSD6 (378), INPP1 (492)
2	10,426,413	10,432,722	FSIP2 (130)
6	84,171,205	8,4195,787	TMPRSS11F (−381), TMPRSS11BNL (−259), NAP1L1 (−253), TMPRSS11E (−177), YTHDC1 (−31), UGT2B10 (248), MGC152010 (478)
8	11,601,528	11,605,893	SCARA3 (−442), CLU (−407), TMEM215 (−266), NDUFB6 (−81), TOPORS (−69), DDX58 (0), ACO1 (6)
22	10,225,287	10,229,539	ARPP21 (−467), STAC (0), LRRFIP2 (118), MLH1 (228), EPM2AIP1 (322), TRANK (−362)

a QTLs with no gene located within a 0.5 Mb window of their boundaries are not included in the table.

### Carcass Fat

**FIGURE 3:**
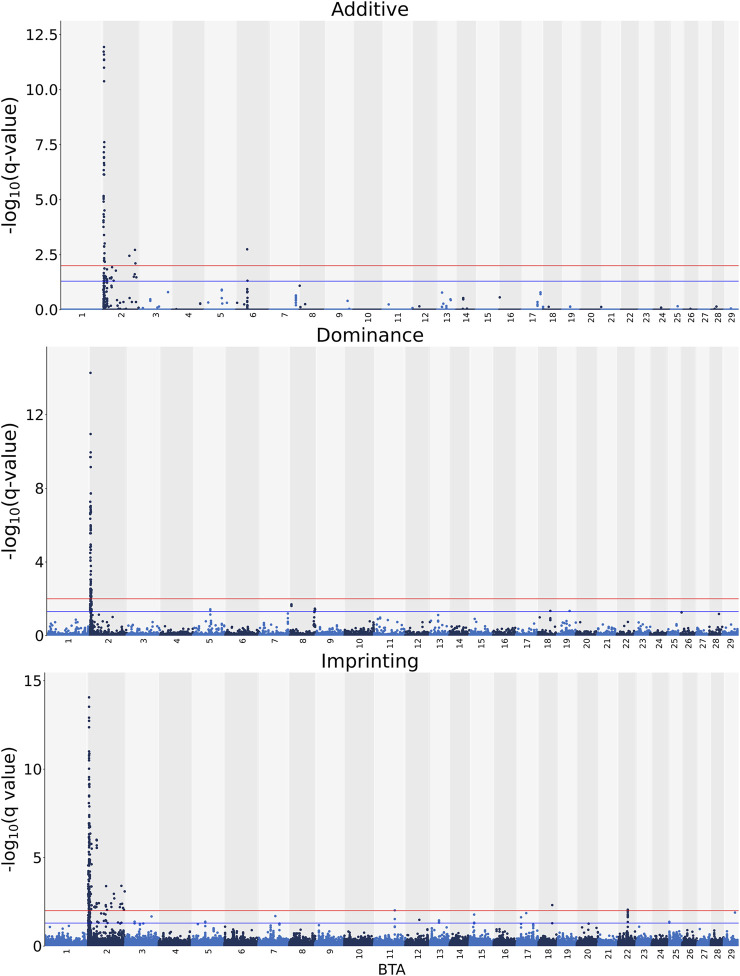
Manhattan plots of the −log_10_ (q values) for additive, dominance and imprinting associations with carcass fat from each *Bos taurus* (BTA) chromosome. Blue and red lines are the thresholds for suggestive (0.01 < q value ≤0.05) and significant (q values ≤0.01) SNPs, respectively.

The additive, dominance and imprinting associations of each SNP of interest and CF are shown in Figure 3, while the QTL regions for CF and the name of genes co-located with each region are in Tables 5 and 6, respectively. Of the 618,837 SNPs included in the association analysis, 218 SNPs demonstrated a significant imprinting association with CF. Of these SNPs, 212 and four were located on BTA 2 and 22, respectively, with single SNPs on BTA 11 and 18 also demonstrating significant imprinting association with CF. An additional 98 SNPs, located on 11 different chromosomes, demonstrated suggestive imprinting associations with CF. The 316 SNPs demonstrating an imprinting association with CF collapsed into 24 distinct QTL regions on BTA 2, as well as into a single QTL region on BTA 5 and two QTL regions on BTA 22. While no currently known imprinted gene resided within a 0.5 Mb window of the QTL regions in question, the lead SNPs of the defined QTL regions did include SNPs located within the genes: OCA2, NIPA1, TUBGCP5, IMP4, SAP130, MYO7B, INPP1, SRGAP1, and TAFA4.

**TABLE 5:** Chromosome (BTA), start and end position (bp), and the number of significant and suggestive single nucleotide polymorphisms (SNPs) within the imprinted QTLs associated with carcass fat, and the SNP name, position, imprinting effect, *p*-value, nearest gene within 0.5 Mb and annotation of the lead SNP.

BTA	QTL region			Most strongly associated SNP in each QTL					
	Start	End	SNPs	SNP name	Position	Effect	*p*-value	Gene	Annotation
2	426,893	584,198	4	rs110346519	560,153	−0.048	6.50 × 10^−8^	OCA2	Intron
2	897,737	999,035	10	rs43598124	970,896	0.051	1.01 × 10^−8^	NIPA1	Upstream
2	1,001,853	1,005,580	3	rs136216394	1,001,853	−0.050	1.51 × 10^−8^	NIPA1	Intron
2	1,283,089	1,283,778	2	rs109730024	1,283,778	−0.038	1.46 × 10^−5^	TUBGCP5	Intron
2	1,319,335	1,323,116	2	rs43287962	1,319,335	0.038	1.27 × 10^−5^	IMP4	Intron
2	2,389,133	2,479,032	4	rs110703910	2,423,609	−0.050	1.08 × 10^−7^	PLEKHB2	Downstream
2	2,486,005	2,555,551	4	rs135620549	2,555,551	−0.065	6.32 × 10^−10^	-	Intergenic
2	2,589,767	2,917,070	15	rs133122826	2,589,767	−0.071	4.20 × 10^−13^	-	Intergenic
2	3,020,151	3,038,367	7	rs109992924	3,031,217	−0.056	4.31 × 10^−8^	-	Intergenic
2	3,044,540	3,061,170	2	rs136731081	3,044,540	−0.050	1.90 × 10^−7^	-	Intergenic
2	3,227,977	3,311,963	10	rs109083210	3,311,963	−0.072	1.37 × 10^−14^	-	Intergenic
2	3,325,739	3,716,374	39	rs137583453	3,496,672	−0.084	1.49 × 10^−20^	-	Intergenic
2	3,789,255	3,824,559	6	rs110334211	3,816,318	−0.060	1.73 × 10^−9^	HS6ST1	Upstream
2	3,864,717	3,917,408	7	rs109255152	3,909,497	0.058	6.76 × 10^−11^	HS6ST1	Upstream
2	3,941,047	3,961,490	5	rs136805866	3,945,897	0.044	1.69 × 10^−6^	HS6ST1	Upstream
2	4,095,906	4,104,393	4	rs135686370	4,100,735	0.067	2.15 × 10^−12^	HS6ST1	Upstream
2	4,232,890	4,405,833	26	rs109937496	4,405,833	0.075	2.49 × 10^−16^	UGGT1	Downstream
2	4,424,778	4,427,108	3	rs137671107	4,424,778	−0.074	2.24 × 10^−16^	UGGT1	Downstream
2	4,442,970	4,525,365	16	rs43288126	4,470,902	−0.061	1.22 × 10^−11^	SAP130	Intron
2	4,970,559	4,975,993	7	rs134723626	4,975,993	−0.040	4.08 × 10^−6^	MYO7B	Intron
2	5,919,765	5,923,766	2	rs135394877	5,919,765	0.061	2.40 × 10^−8^	INPP1	Upstream
2	5,930,467	5,931,880	3	rs109350048	5,931,880	0.061	2.02 × 10^−8^	INPP1	Upstream
2	5,960,560	6,152,011	7	rs134868949	6,148,317	0.060	9.08 × 10^−10^	INPP1	Intron
2	6,256,331	6,258,201	2	rs109161021	6,258,201	0.043	8.44 × 10^−7^	MSTN	Upstream
5	49,598,490	49,602,780	2	rs136591812	49,598,490	−0.041	2.09 × 10^−5^	SRGAP1	Intron
22	32,758,667	32,759,632	2	rs133992067	32,759,632	−0.041	1.04 × 10^−5^	TAFA4	Intron
22	32,769,829	32,800,425	6	rs110953616	32,769,829	−0.043	3.14 × 10^−6^	TAFA4	Intron

**TABLE 6:** Chromosome (BTA) and start and end position (bp) of each imprinted QTL regions defined for carcass fat, as well as the name of genes located within a 0.5 Mb window of each region [distance (in Kb) from the lead SNP of each region in parenthesis]^a^.

BTA	Start	End	Genes
2	426,893	584,198	LGSN (−44), OCA2 (0), HERC2 (136), NIPA1 (405), NIPA2 (460), CYFIP1 (483)
2	897,737	999,035	OCA2 (−193), HERC2 (0), NIPA1 (0), NIPA2 (46), CYFIP1 (68), TUBGCP5 (234), CCDC115 (315), IMP4 (319), PTPN18 (324), AMER3 (475)
2	1,001,853	1,005,580	OCA2 (−296), HERC2 (−50), NIPA1 (0), NIPA2 (39), CYFIP1 (61), TUBGCP5 (227), CCDC115 (309), IMP4 (313), PTPN18 (317), AMER3 (468)
2	1,283,089	1,283,778	HERC2 (−331), NIPA1 (−249), NIPA2 (−218), CYFIP1 (−108), TUBGCP5 (0), CCDC115 (30), IMP4 (35), PTPN18 (39), AMER3 (190), ARHGEF4 (396)
2	1,319,335	1,323,116	HERC2 (−367), NIPA1 (−285), NIPA2 (−254), CYFIP1 (−144), TUBGCP5 (−35), CCDC115 (−1), IMP4 (0), PTPN18 (0), AMER3 (150), ARHGEF4 (356)
2	2,389,133	2,479,032	FAM168B (−483), PLEKHB2 (−419)
2	3,325,739	3,716,374	HS6ST1 (470)
2	3,789,255	3,824,559	HS6ST1 (363), UGGT1 (475)
2	3,864,717	3,917,408	HS6ST1 (270), UGGT1 (383)
2	3,941,047	3,961,490	HS6ST1 (225), UGGT1 (339), SAP130 (493)
2	4,095,906	4,104,393	HS6ST1 (83), UGGT1 (196), SAP130 (351), AMMECR1L (497)
2	4,232,890	4,405,833	HS6ST1 (−6), UGGT1 (0), SAP130 (49), AMMECR1L (195), POLR2D (218), WDR33 (271), SFT2D3 (385), LIMS2 (436), GPR17 (467), MYO7B (481)
2	4,424,778	4,427,108	HS6ST1 (−197), UGGT1 (−26), SAP130 (28), AMMECR1L (174), POLR2D (196), WDR33 (250), SFT2D3 (363), LIMS2 (415), GPR17 (446), MYO7B (460)
2	4,442,970	4,525,365	HS6ST1 (−216), UGGT1 (−44), SAP130 (0), AMMECR1L (76), POLR2D (98), WDR33 (152), SFT2D3 (265), LIMS2 (317), GPR17 (348), MYO7B (362)
2	4,970,559	4,975,993	SAP130 (−441), AMMECR1L (−350), POLR2D (−333), WDR33 (−185), SFT2D3 (−180), LIMS2 (−83), GPR17 (−92), MYO7B (0), IWS1 (55), PROC (126), MAP3K2 (223), ERCC3 (273), CYP27C1 (321), BIN1 (442)
2	5,919,765	5,923,766	BIN1 (−445), NAB1 (−454), NEMP2 (−236), MFSD6 (−37), INPP1 (10), HIBCH (57), MSTN (355)
2	5,930,467	5,931,880	BIN1 (−455), NAB1 (−247), NEMP2 (−120), MFSD6 (−49), INPP1 (3), HIBCH (49), MSTN (347)
2	5,960,560	6,152,011	BIN1 (−486), NAB1 (−277), NEMP2 (−150), MFSD6 (−79), INPP1 (0), HIBCH (0), MSTN (20), PMS1 (308), ORMDL1 (420), OSGEPL1 (441), ANKAR (454)
2	6,256,331	6,258,201	NEMP2 (−446), MFSD6 (−374), INPP1 (−285), HIBCH (−127), MSTN (29), PMS1 (202), ORMDL1 (314), OSGEPL1 (335), ANKAR (348), ASND1 (407)
5	49,598,490	49,602,780	GNS (−495), RASSF3 (−408), TBK1 (−283), XPOT (−131), SPGAP1 (0), RXYLT1 (317)
22	32,758,667	32,759,632	FRMD4B (−377), LM0D3 (−325), ARL6IP5 (−301), UBA3 (−267), TMF1 (−233), EOGT (−188), TAFA4 (0), TAFA4 (207)
22	32,769,829	32,800,425	FRMD4B (−388), LM0D3 (−336), ARL6IP5 (−311), UBA3 (−278), TMF1 (−244), EOGT (−194), TAFA4 (0), TAFA4 (166)

a QTLs with no gene located within a 0.5 Mb window of their boundaries are not included in the table.

## Discussion

The term epigenetics or, in other words, the manifestation of divergent phenotypes from the same genotype, can be attributed to Conrad Waddington (Waddington, 1939). An example of this phenomenon occurs in female ants whom, with little to no differences in their genome, have the potential to manifest very diverse phenotypes, namely that of a queen, a major worker, or a minor worker (Chittka et al., 2012). This specific example of epigenetics was observed by Darwin who described it as a “special difficulty, which at first appeared to me insuperable, and actually fatal to the whole theory [of evolution by natural selection]” (Darwin, 1859). Nevertheless, research has since discovered that such phenomena are not, in Darwin’s words, insuperable, but rather the result of various biological mechanisms, primarily DNA methylation and histone modifications, that underline the epigenome (MacDonald, 2012; Ibeagha-Awemu and Zhao, 2015). These mechanisms can underline, among other things, the epigenetic phenomenon known as genomic imprinting (MacDonald, 2012). In the context of animal breeding, information regarding SNPs demonstrating imprinting associations with a trait of interest can be incorporated into genomic evaluations (Varona et al., 2018) to improve the accuracy associated with the breeding values derived from such evaluations (Nishio and Satoh, 2015). Furthermore, knowledge of the loci at which imprinting associations exist for carcass metrics could be useful for mating advice programs to exploit (or avoid) the effects of genomic imprinting in the subsequent offspring. Such mating advice programs could perhaps use a platform similar to that described by Upperman et al. (2019), which is used in genotyped populations to reduce the risk of progeny/embryos resulting from a given mating inheriting a known recessive lethal gene. While this knowledge could be useful to all beef matings, such knowledge could perhaps be particularly useful in dairy-beef matings; an ever-increasing proportion of animals bred for beef production are the progeny of specialized dairy dams and specialized beef sires. Therefore, dairy-beef animals inherit their paternal- and maternal-derived alleles at each locus from genomes comprising allele frequencies and mutations that are the result of selection for traits important to beef and dairy production, respectively.

The presence of imprinting genes in cattle has been previously documented, with 20–30 imprinted genes currently validated in the *Bos taurus* genome (Morison et al., 2001; O'Doherty et al., 2015). Furthermore, the presence of SNPs demonstrating imprinting association with carcass traits have been previously reported in cattle (Magee et al., 2010a; Magee et al., 2010b; Imumorin et al., 2011), although no such analysis has yet been conducted using high-density genotype data, or using such a large group of cattle as that used in the present study. In mice, the number of currently known imprinted genes is approximately 80 (Wolf et al., 2008b), although the number of genes predicted to be imprinted in the *Mus musculus* genome is as high as 600 (Luedi et al., 2005). Wolf et al. (2008b), who detected numerous novel imprinted loci in mice, attributed the discrepancy between the number of known and predicted imprinted loci in mice to the use of a small number of SNPs, perhaps from a specific region of the genome, as opposed to the analyses of high-density or whole-genome SNP data. Similarly, Magee et al. (2010a), Magee et al. (2010b) and Imumorin et al. (2011), attempted to locate imprinted SNPs for carcass traits, using just seven SNPs, 17 SNPs and a linkage map constructed from 357 microsatellites, respectively, in their analysis. Compared to the locations of currently known imprinted genes in cattle (www.geneimprint.com), novel loci demonstrating imprinting associations were detected in the present study. Nonetheless, the imprinting effects (i.e., the difference between the mean of the two phased heterozygous genotypes of a given locus and the mid-point of the two means of the corresponding homozygous genotypes) associated with the novel loci detected in the present study were biologically small. For example, the largest imprinting effect estimated for the different lead SNPs associated with CW, CC, and CF in the present study were equivalent to 5.0%, 7.1%, and 8.9% of the respective genetic standard deviations estimated for the traits of interest by Kenny et al. (2020a).

As per Wolf et al. (2008a), based on the effects associated with the lead SNPs of the QTL regions defined in the present study, the imprinting mechanism associated with the majority of the lead SNPs for CW and CC was maternal imprinting, while that for the majority of the lead SNPs associated with CF was paternal imprinting. Maternal imprinting refers to the full expression of the paternal allele only, while paternal imprinting refers to the full expression of the maternal allele only. The mechanisms detected in the present study are corroborated by the parental tug-of-war hypothesis outlined by Moore and Haig (1991). This hypothesis states that paternally expressed genes (i.e., maternal imprinting) promote growth in offspring, while maternal expression (i.e., paternal imprinting) inhibit growth in offspring. In line with the hypothesis and the mechanisms detected for each trait in the present study, positive genetic correlations have been previously reported between growth traits and both CW and CC in cattle (Francoise et al., 1973; Choi et al., 2015). Furthermore, negative genetic correlations have been previously reported between body fat measures and growth traits in cattle (Arnold et al., 1991).

### Link Between Discovered Imprinted Regions and Known Imprinted Regions

The presence of imprinting associations with the carcass traits of interest were detected on 19 different chromosomes in the present study. Of these, the vast majority of the SNPs detected to have imprinting associations were located on BTA 2; this is despite the fact that no imprinted genes have, to the best of our knowledge, yet been validated on BTA 2. Nonetheless, the presence of novel imprinting regions for carcass traits, namely CW and kidney, pelvic and heart fat, on BTA 2 was previously documented in cattle by Imumorin et al. (2011). Similar to the present study, Imumorin et al. (2011) advocates the need for further exploration on this chromosome. A bovine ortholog of a gene known to be imprinted in both humans and mice (Gregg et al., 2010) was located within the imprinted QTL regions detected for carcass traits on BTA 2 by Imumorin et al. (2011). While the gene, IWS1, has yet to be documented as imprinted in cattle, it also resided within 0.5 Mb of QTL regions identified in the present study. With regard to the other 18 chromosomes where imprinting associations were detected in the present study, seven also contain known imprinted genes in the *Bos taurus* genome; these included BTA 3, 4, 6, 13, 18, 21, and 25. Of the SNPs demonstrating imprinting associations located on these chromosomes, the nearest SNP demonstrating an imprinting association to a known imprinted gene (i.e., rs41649705) was 2.7 Mb downstream from ZNF597 on BTA 25.

While not all known imprinted cattle genes had intronic SNPs included in the present study (i.e., DIRAS3, NAP1L5, MAGEL2, LOC100849023, DIO3, DLK1, and ZNF597), all known imprinted genes did have at least one SNP within a 50 Kb window of their location included in the analyses. Of the SNPs located within these 50 Kb windows, the average and standard deviation of their minor allele frequencies were 0.30 and 0.13, respectively. All q values associated with the imprinting effects estimated for SNPs within a 50 Kb window of a known imprinted gene were ≥0.99 for CW (*p* values ≥0.628), ≥0.56 for CC (*p* values ≥0.002), and ≥0.59 for CF (*p* values ≥0.007); extending the window to 0.5 Mb, the q values associated with the imprinting effects estimated for SNPs within the larger windows were all ≥0.86 for CW (*p* values ≥0.001), ≥0.56 for CC (*p* values ≥0.002), and ≥0.16 for CF (*p* values ≥0.0001). This signifies that, based on the analysis conducted herein, even if a less conservative genome-wide significance threshold was used, no currently known imprinted genes demonstrated imprinting associations with the carcass traits evaluated. The relatively large dataset of animals employed for this analysis should ensure that the analysis conducted had the necessary power to detect any imprinting associations for the carcass traits, should they exist. Similarly, Imumorin et al. (2011) in their analysis of carcass traits did not detected imprinting associations for carcass traits either within or nearby any known imprinted genes.

While no genes co-located with QTLs detected in the present study were currently known imprinted genes, these co-located genes included genes that have previously been recognized as major genes associated, not only, with the carcass traits of interest, but also other traits of importance to beef production systems. One example of this is the gene MSTN (which was upstream from detected QTLs in the present study), with Purfield et al. (2019a) estimating, for example, that the Q204X mutation of the MSTN gene accounted for 1.2, 1.1, and 6.0% of the genetic variance in CW, CF and CC, respectively in Charolais cattle. In addition, Purfield et al. (2020) estimated that the same MSTN mutation accounted for 5.1% of the genetic variance associated with calving difficulty in Charolais cattle. Other co-located genes detected herein to have imprinting associations that have also been previously detected to have additive associations with the traits of interest include: HS6ST1, BIN1, WDR33, and HERC2 (Purfield et al., 2019a; Kenny et al., 2022). Therefore, results herein further suggest the need to validate the imprinting status of novel imprinting genes detected in this study, as well as the potential for more informed breeding decisions based on the imprinting status of major genes, such as MSTN.

## Conclusion

Of the 618,837 SNPs included in the association analyses, 24 SNPs, 339 SNPs, and 316 SNPs, spread across 19 different chromosomes, demonstrated some form of imprinting association (i.e., significant or suggestive) with CW, CC, and CF, respectively. The fact that all imprinting associations detected for the carcass traits of interest were at novel loci warrants further investigation in these regions, particularly, BTA 2 where the vast majority of the imprinting associations were detected. Nonetheless, the data used to detect these imprinting associations comprised high-density genotype data from a relatively large group of cattle, which should ensure the analyses conducted had the power to detect imprinting associations, should they exist. Knowledge of loci at which imprinting associations are present for the carcass traits of interest could be incorporated into genomic evaluations in an attempt to improve the accuracy of such evaluations for these traits. Furthermore, this knowledge could be incorporated into mate allocation programs to exploit (or avoid) the effects of genomic imprinting in potential offspring.

## Data Availability

The data analyzed in this study is subject to the following licenses/restrictions: Individual genotype and phenotype data used in this study are managed by a third party, the Irish Cattle Breeding Federation (ICBF). Reasonable requests for data can be made to the Irish Cattle Breeding Federation, Highfield House, Shinagh, Bandon, Co. Cork, Ireland (email: query@icbf.com; website: https://www.icbf.com/). Requests to access these datasets should be directed to query@icbf.com.

## Abbreviations

BTA, Bos taurus chromosome; CC, carcass conformation; CF, carcass fat; CW, carcass weight; QTL, quantitative trait loci; SNPs, single nucleotide polymorphisms.

## References

[B1] Arnold J. W. Bertrand J. K. Benyshek L. L. Ludwig C. (1991). Estimates of Genetic Parameters for Live Animal Ultrasound, Actual Carcass Data, and Growth Traits in Beef Cattle. J. Animal Sci. 69 (3), 985–992. 10.2527/1991.693985x 2061268

[B2] Aulchenko Y. S. De Koning D.-J. Haley C. (2007a). Genomewide Rapid Association Using Mixed Model and Regression: a Fast and Simple Method for Genomewide Pedigree-Based Quantitative Trait Loci Association Analysis. Genetics 177 (1), 577–585. 10.1534/genetics.107.075614 17660554 PMC2013682

[B3] Aulchenko Y. S. Ripke S. Isaacs A. Van Duijn C. M. (2007b). GenABEL: an R Library for Genome-wide Association Analysis. Bioinformatics 23 (10), 1294–1296. 10.1093/bioinformatics/btm108 17384015

[B4] Cattanach B. M. Kirk M. (1985). Differential Activity of Maternally and Paternally Derived Chromosome Regions in Mice. Nature 315 (6019), 496–498. 10.1038/315496a0 4000278

[B5] Chittka A. Wurm Y. Chittka L. (2012). Epigenetics: the Making of Ant Castes. Curr. Biol. 22 (19), R835–R838. 10.1016/j.cub.2012.07.045 23058801

[B6] Choi T. J. Alam M. Cho C. I. Lee J. G. Park B. Kim S. (2015). Genetic Parameters for Yearling Weight, Carcass Traits, and Primal-Cut Yields of Hanwoo Cattle. J. animal Sci. 93 (4), 1511–1521. 10.2527/jas.2014-7953 26020173

[B7] Darwin C. (1859). On the Origin of Species by Means of Natural Selection. London: John Murray.

[B8] Durinck S. Spellman P. T. Birney E. Huber W. (2009). Mapping Identifiers for the Integration of Genomic Datasets with the R/Bioconductor Package biomaRt. Nat. Protoc. 4, 1184–1191. 10.1038/nprot.2009.97 19617889 PMC3159387

[B9] Essl A. Voith K. (2002). Genomic Imprinting Effects on Dairy- and Fitness-Related Traits in Cattle. J. Anim. Breed. Genet. 119 (3), 182–189. 10.1046/j.1439-0388.2002.00334.x

[B10] Francoise J. J. Vogt D. W. Nolan J. C. Jr (1973). Heritabilities of and Genetic and Phenotypic Correlations Among Some Economically Important Traits of Beef Cattle. J. Animal Sci. 36 (4), 635–639. 10.2527/jas1973.364635x

[B11] García-Ruiz A. Cole J. B. VanRaden P. M. Wiggans G. R. Ruiz-López F. J. Van Tassell C. P. (2016). Changes in Genetic Selection Differentials and Generation Intervals in US Holstein Dairy Cattle as a Result of Genomic Selection. Proc. Natl. Acad. Sci. 113 (28), E3995–E4004. 10.1073/pnas.1519061113 27354521 PMC4948329

[B12] Georges M. Charlier C. Cockett N. (2003). The Callipyge Locus: Evidence for the Trans Interaction of Reciprocally Imprinted Genes. Trends Genet. 19 (5), 248–252. 10.1016/s0168-9525(03)00082-9 12711215

[B13] González-Recio O. Toro M. A. Bach A. (2015). Past, Present and Future of Epigenetics Applied to Livestock Breeding. Front. Genet. 6, 305. 10.3389/fgene.2015.00305 26442117 PMC4585102

[B14] Graser H.-U. Tier B. Johnston D. J. Barwick S. A. (2005). Genetic Evaluation for the Beef Industry in Australia. Aust. J. Exp. Agric. 45 (8), 913–921. 10.1071/ea05075

[B15] Gregg C. Zhang J. Weissbourd B. Luo S. Schroth G. P. Haig D. (2010). High-Resolution Analysis of Parent-Of-Origin Allelic Expression in the Mouse Brain. Science 329 (5992)**,** 643–648. 10.1126/science.1190830 20616232 PMC3005244

[B16] Guo X. Christensen O. F. Ostersen T. Wang Y. Lund M. S. Su G. (2016). Genomic Prediction Using Models with Dominance and Imprinting Effects for Backfat Thickness and Average Daily Gain in Danish Duroc Pigs. Genet. Sel. Evol. 48 (1), 67–69. 10.1186/s12711-016-0245-6 27623617 PMC5022243

[B17] Havenstein G. Ferket P. Qureshi M. (2003). Growth, Livability, and Feed Conversion of 1957 versus 2001 Broilers when Fed Representative 1957 and 2001 Broiler Diets. Poult. Sci. 82 (10), 1500–1508. 10.1093/ps/82.10.1500 14601725

[B18] Ibeagha-Awemu E. M. Zhao X. (2015). Epigenetic Marks: Regulators of Livestock Phenotypes and Conceivable Sources of Missing Variation in Livestock Improvement Programs. Front. Genet. 6, 302. 10.3389/fgene.2015.00302 26442116 PMC4585011

[B19] Imumorin I. G. Kim E.-H. Lee Y.-M. De Koning D.-J. Van Arendonk J. A. De Donato M. (2011). Genome Scan for Parent-Of-Origin QTL Effects on Bovine Growth and Carcass Traits. Front. Gene. 2, 44. 10.3389/fgene.2011.00044 PMC3268597 22303340

[B20] Inoue K. Inoue Y. Oe T. Nishimura M. (2021). Genomic Imprinting Variances of Beef Carcass Traits and Physiochemical Characteristics in Japanese Black Cattle. Animal Sci. J. 92 (1), e13504. 10.1111/asj.13504 33458906

[B21] Kenny D. Judge M. M. Sleator R. D. Murphy C. P. Evans R. D. Berry D. P. (2020a). The Achievement of a Given Carcass Specification Is under Moderate Genetic Control in Cattle. J. Anim. Sci. 98 (6), skaa158. 10.1093/jas/skaa158 32459312 PMC7299552

[B22] Kenny D. Murphy C. P. Sleator R. D. Judge M. M. Evans R. D. Berry D. P. (2020b). Animal-Level Factors Associated with the Achievement of Desirable Specifications in Irish Beef Carcasses Graded Using the EUROP Classification System. J. Anim. Sci. 98 (7), skaa191. 10.1093/jas/skaa191 32516387 PMC7333216

[B23] Kenny D. Carthy T. R. Murphy C. P. Sleator R. D. Evans R. D. Berry D. P. (2022). The Association between Genomic Heterozygosity and Carcass Merit in Cattle. Front. Genet. 13, 789270. 10.3389/fgene.2022.789270 35281838 PMC8908906

[B24] Luedi P. P. Hartemink A. J. Jirtle R. L. (2005). Genome-wide Prediction of Imprinted Murine Genes. Genome Res. 15 (6), 875–884. 10.1101/gr.3303505 15930497 PMC1142478

[B25] MacDonald W. A. (2012). Epigenetic Mechanisms of Genomic Imprinting: Common Themes in the Regulation of Imprinted Regions in Mammals, Plants, and Insects. Genet. Res. Int. 2012, 585024. 10.1155/2012/585024 22567394 PMC3335465

[B26] Magee D. A. Sikora K. M. Berkowicz E. W. Berry D. P. Howard D. J. Mullen M. P. (2010b). DNA Sequence Polymorphisms in a Panel of Eight Candidate Bovine Imprinted Genes and Their Association with Performance Traits in Irish Holstein-Friesian Cattle. BMC Genet. 11 (1), 93–15. 10.1186/1471-2156-11-93 20942903 PMC2965127

[B27] Magee D. A. Berry D. P. Berkowicz E. W. Sikora K. M. Howard D. J. Mullen M. P. (2010a). Single Nucleotide Polymorphisms within the Bovine DLK1-DIO3 Imprinted Domain Are Associated with Economically Important Production Traits in Cattle. J. Hered. 102 (1), 94–101. 10.1093/jhered/esq097 20817761

[B28] Moore T. Haig D. (1991). Genomic Imprinting in Mammalian Development: a Parental Tug-of-War. Trends Genet. 7 (2), 45–49. 10.1016/0168-9525(91)90230-n 2035190

[B29] Morison I. M. Paton C. J. Cleverley S. D. (2001). The Imprinted Gene and Parent-of-Origin Effect Database. Nucleic acids Res. 29 (1), 275–276. 10.1093/nar/29.1.275 11125110 PMC29803

[B30] Neugebauer N. Räder I. Schild H. J. Zimmer D. Reinsch N. (2010). Evidence for Parent-of-Origin Effects on Genetic Variability of Beef Traits. J. animal Sci. 88 (2), 523–532. 10.2527/jas.2009-2026 19854988

[B31] Nishio M. Satoh M. (2015). Genomic Best Linear Unbiased Prediction Method Including Imprinting Effects for Genomic Evaluation. Genet. Sel. Evol. 47 (1), 32–10. 10.1186/s12711-015-0091-y 25928098 PMC4404063

[B32] O’Doherty A. M. MacHugh D. E. Spillane C. Magee D. A. (2015). Genomic Imprinting Effects on Complex Traits in Domesticated Animal Species. Front. Genet. 6, 156. 10.3389/fgene.2015.00156 25964798 PMC4408863

[B33] Pabiou T. Fikse W. F. Amer P. R. Cromie A. R. Näsholm A. Berry D. P. (2012). Genetic Relationships between Carcass Cut Weights Predicted from Video Image Analysis and Other Performance Traits in Cattle. Animal 6 (9), 1389–1397. 10.1017/s1751731112000705 22717237

[B34] Purfield D. C. Evans R. D. Berry D. P. (2020). Breed- and Trait-specific Associations Define the Genetic Architecture of Calving Performance Traits in Cattle. J. Anim. Sci. 98 (5), skaa151. 10.1093/jas/skaa151 32365208 PMC7247537

[B35] Purfield D. C. Evans R. D. Berry D. P. (2019a). Reaffirmation of Known Major Genes and the Identification of Novel Candidate Genes Associated with Carcass-Related Metrics Based on Whole Genome Sequence within a Large Multi-Breed Cattle Population. BMC Genomics 20 (1), 720. 10.1186/s12864-019-6071-9 31533623 PMC6751660

[B36] Purfield D. C. Evans R. D. Carthy T. R. Berry D. P. (2019b). Genomic Regions Associated with Gestation Length Detected Using Whole-Genome Sequence Data Differ between Dairy and Beef Cattle. Front. Genet. 10, 1068. 10.3389/fgene.2019.01068 31749838 PMC6848454

[B37] Reik W. Walter J. (2001). Genomic Imprinting: Parental Influence on the Genome. Nat. Rev. Genet. 2 (1), 21–32. 10.1038/35047554 11253064

[B38] Ring S. C. McCarthy J. Kelleher M. M. Doherty M. L. Berry D. P. (2018). Risk Factors Associated with Animal Mortality in Pasture-Based, Seasonal-Calving Dairy and Beef Herds. J. Animal Sci. 96 (1), 35–55. 10.1093/jas/skx072 PMC6140972 29385481

[B39] Sargolzaei M. Chesnais J. P. Schenkel F. S. (2014). A New Approach for Efficient Genotype Imputation Using Information from Relatives. BMC Genomics 15 (1), 478–512. 10.1186/1471-2164-15-478 24935670 PMC4076979

[B40] Storey J. D. Tibshirani R. (2003). Statistical Significance for Genomewide Studies. Proc. Natl. Acad. Sci. U.S.A. 100 (16), 9440–9445. 10.1073/pnas.1530509100 12883005 PMC170937

[B41] Twomey A. J. Cromie A. R. McHugh N. Berry D. P. (2020). Validation of a Beef Cattle Maternal Breeding Objective Based on a Cross-Sectional Analysis of a Large National Cattle Database. J. Anim. Sci. 98 (11), skaa322. 10.1093/jas/skaa322 33011772 PMC7751150

[B42] Upperman L. R. Kinghorn B. P. MacNeil M. D. Van Eenennaam A. L. (2019). Management of Lethal Recessive Alleles in Beef Cattle through the Use of Mate Selection Software. Genet. Sel. Evol. 51 (1), 36. 10.1186/s12711-019-0477-3 31382878 PMC6683482

[B43] VanRaden P. M. (2008). Efficient Methods to Compute Genomic Predictions. J. dairy Sci. 91 (11), 4414–4423. 10.3168/jds.2007-0980 18946147

[B44] Varona L. Legarra A. Toro M. A. Vitezica Z. G. (2018). Non-Additive Effects in Genomic Selection. Front. Genet. 9, 78. 10.3389/fgene.2018.00078 29559995 PMC5845743

[B45] Waddington C. H. (1939). Introduction to Modern Genetics. London: Allen & Unwin.

[B46] Wiggans G. R. Misztal I. Van Vleck L. D. (1988). Implementation of an Animal Model for Genetic Evaluation of Dairy Cattle in the United States. J. Dairy Sci. 71, 54–69. 10.1016/s0022-0302(88)79979-8 3397426

[B47] Wolf J. B. Cheverud J. M. Roseman C. Hager R. (2008a). Genome-wide Analysis Reveals a Complex Pattern of Genomic Imprinting in Mice. PLoS Genet. 4 (6), e1000091. 10.1371/journal.pgen.1000091 18535661 PMC2390766

[B48] Wolf J. B. Hager R. Cheverud J. M. (2008b). Genomic Imprinting Effects on Complex Traits: a Phenotype-Based Perspective. Epigenetics 3 (6), 295–299. 10.4161/epi.3.6.7257 19029803

[B49] Xu Y. Liu X. Fu J. Wang H. Wang J. Huang C. (2020). Enhancing Genetic Gain through Genomic Selection: from Livestock to Plants. Plant Commun. 1 (1), 100005. 10.1016/j.xplc.2019.100005 33404534 PMC7747995

