# Supplementary material for: Detection of Genomic Imprinting for Carcass Traits in Cattle Using Imputed High-Density Genotype Data
Source: Front Genet. 2022 Jul 15;13:951087. doi: 10.3389/fgene.2022.951087 (PMC9334527; doi:10.3389/fgene.2022.951087)
Supplement: Supplementary file 1 [file Table1.DOCX]

**Supplementary material**

**Title: Detection of genomic imprinting for carcass traits in cattle using imputed high-density genotype data**

**Authors:** David Kenny, Roy D. Sleator, Craig P. Murphy, Ross D. Evans and Donagh P. Berry

**Supplementary Table S1** Coding of the four distinct phased genotypes^1^ to represent additive, dominance and imprinting effects

| Genotypes | AA | AB | BA | BB |
| --- | --- | --- | --- | --- |
| Additive | 0 | 1 | 1 | 2 |
| Dominance | 0 | 1 | 1 | 0 |
| Imprinting | 0 | -1 | 1 | 0 |

^1^the first allele of the heterozygote genotype represents the allele inherited from the sire, while the second represents that inherited from the dam
